# Supplementary material for: Are carbon emissions trading and green financial instruments synergistic? -Comprehensive quantitative research based on content analysis
Source: PLoS One. 2024 Mar 7;19(3):e0298601. doi: 10.1371/journal.pone.0298601 (PMC10919601; doi:10.1371/journal.pone.0298601)
Supplement: S1 Data — (ZIP) [file pone.0298601.s001.zip › Data/地级市Z_F工作报告.zip!/─■╧─/╓╨╬└╩╨/└┤╫╘╣1⁄2╓┌║┼í╛┬φ┐╦╩2╛▌═°í┐_2006.docx]

各位代表：

现在，我代表市人民政府，向大会作政府工作报告，请予审议，并请政协委员和其他列席人员提出意见。

一、2005年工作回顾

2005年，市人民政府在自治区党委、政府和市委的正确领导下，以党的十六大和十六届四中、五中全会精神为指导，发扬激情创业、热情干事的精神，克服严重干旱、电价上涨、银根地根紧缩等诸多困难，团结和带领全市人民加快推进新型工业化、城市化、农业产业化和旅游特色化，全面完成了市一届人大二次会议确定的各项目标任务。预计全市地区生产总值完成58.34亿元，同比增长12%。其中：第一产业14.06亿元，同比增长2.8%；第二产业25.85亿元，同比增长15.2%；第三产业18.43亿元，同比增长10.3%。城区地区生产总值30.6亿元，同比增长13.5%。地方财政收入3.55亿元，可比增长16.3%；其中市本级财政收入2.1亿元，可比增长20.5%。全社会固定资产投资54亿元。城区全社会固定资产投资26亿元，同比增长16.2%。城镇居民人均可支配收入7200元，同比增长9.2%。农民人均纯收入2500元，同比增长5.9%。城镇登记失业率3.7%。人口自然增长率11.5‰。

工业经济快速增长，综合实力大幅提升。全市工业总产值预计完成57亿元，同比增长23.5%；工业增加值16.8亿元，同比增长22%。全市规模以上企业达到75家，产值、增加值和利税分别为45亿元、14亿元和3.6亿元，同比分别增长26%、25%和15%。产值超亿元的企业12家、超5亿元的2家。各工业园区和工业基地入园企业累计达到79家，建成投产61家，产值和增加值分别占全市工业总产值、增加值的23.6%和24.4%。全市共引进各类项目135个，到位资金14.8亿元。美利纸业30万吨涂布白卡纸、10万吨杨木化机浆和昊丰伟业生铁、赢海集团金属镁、大河机床数控珩磨机床等一批重点项目正在加紧建设；宁夏日盛和创业公司电石、科豪陶瓷外墙砖、深圳东部集团浓缩果汁、磊鑫矿业湿法炼铜等项目建成投产。

特色产业不断壮大，农村经济稳步发展。全市农业总产值预计完成26.46亿元，同比增长5.5%。新增枸杞3.1万亩，累计达到16.1万亩；新增设施蔬菜1.33万亩，累计达到5.33万亩；新增压砂地20万亩，累计达到50万亩，形成了以香山、兴仁、鸣沙、白马、喊叫水为主产地的硒砂瓜产业带；新增人工种草10万亩，累计达到131.7万亩。在抓好宣和百万只鸡养殖园区达产达标的同时，建成了永康百万只鸡养殖园区东区，全市鸡、羊、牛、猪饲养量分别达到1320万只、132万只、16.9万头、79万头。发展各类农产品加工企业58家，组建各类流通组织、专业协会49个，年流通额突破6亿元。新建规范各类市场19个。完成了“宣和禽蛋”等8类13个无公害农产品区级认定和“香山硒砂瓜”的商标注册，城区蔬菜进入全国200个创建无公害农产品生产示范基地达标县行列。农业基础设施得到加强，引黄灌区续建配套与节水改造、山区小流域治理和治河治沟工程进展顺利。完成了红三公路二期等工程建设，硬化乡村主干道路83条137.5公里，累计达到267.5公里，乡村交通环境明显改善。

城市建设快速推进，区域优势日益显现。编制完成了新区总体规划和控制性详规。全市共确定新建、续建城市建设项目131个，完成投资15.6亿元。建成了市区中央大道、迎宾大道、平安大道、卫谢南路、应理南路、五葡路等骨干道路，拉大了城市框架。建设了市区“一园”（黄河湿地公园）、“两湖”（新区生态湖、沙坡头生态风情苑南湖）、“三带”（中央大道景观带、滨河大道景观带、彩虹大道景观带）基础工程，完成了中营高速公路中宁至中卫段路基工程和卫宁公路永康段维修，提升了城市品位。实施了城市供排水、集中供热、垃圾无害化处理等工程，有效改善了城市环境质量。建成了一批高档次住宅小区,居民居住条件极大改善。加强城市综合执法和环境整治，城市面貌明显改观。中宁新南街路、裕民路、枸杞花园和海原利民花园等城市重点建设项目进展顺利。

旅游商贸持续繁荣，第三产业快速发展。完成了《中卫旅游发展总体规划》和部分景区规划，充分挖掘大漠黄河、杞乡文化、海原环球大震遗址等旅游资源，旅游基础设施建设和旅游产品开发力度进一步加大，沙坡头、寺口子等旅游景区基础设施建设进展顺利，开发了沙坡头生态风情园等旅游项目，沙漠产品的包装和推出取得突破性进展。沙坡头被评为“中国五大最美的沙漠”之一和“国家水利风景区”，中卫旅游知名度进一步提高。全市各旅游景区（点）接待游客85.9万人次，实现旅游总收入1.9亿元，同比分别增长15.3%和29.4%。商贸服务、餐饮娱乐、交通运输等第三产业快速发展。全社会消费品零售总额达到14.85亿元，同比增长13.3%。

财税金融有效加强，各项改革继续推进。进一步加大税收征管力度，财源建设初显成效。强化财政支出管理，调整优化支出结构，加大农业、社会保障和社会公共事业发展的投入力度，保证了重点建设和重要支出项目的基本需要，财政保障能力明显提高。稳步推进部门预算、国库集中收付、政府采购、镇乡财政管理体制改革，深化农村税费改革，全面取消了农业税。推行了灌区供水体制和水价制度改革。进一步完善了企业法人治理结构，加快了改制企业职工身份置换。完成了镇乡人员定岗分流工作。对所有事业单位重新进行了审核登记，逐步实行编制实名制管理。依法加强了国有资产监管，确保了国有资产保值增值。全面推行项目核准制和备案制，落实了企业投资自主权。建立投资决策责任制，规范了政府投资行为。加强银政、银企合作，金融企业信贷规模不断扩大，服务领域进一步拓展。预计全市金融机构存款余额69亿元，同比增长12.4%；贷款余额65.5亿元，同比增长13.5%。

科教兴市战略深入实施，社会事业全面发展。争取和实施教育项目16个，总投资2038万元，教育基础设施和办学条件得到改善。素质教育不断深化，教育教学质量稳步提高。加快科技进步与技术创新，推动高新技术产业化，企业核心竞争力不断增强。深入实施科技特派员创业行动，科普工作取得新成绩。推进文化体制改革，加强精品创作，规范文化市场秩序，文化事业全面发展。全年广场文艺演出204场次，群众文化丰富多彩。全民健身运动蓬勃开展，“体育下乡、篮球进村”工程深入实施。认真贯彻《公民道德建设实施纲要》，开展了一系列群众性精神文明创建活动，抵制封建迷信活动和邪教渗透取得明显成效。实施农村广播影视公共服务体系建设项目，全市80%的农村群众免费收看到8套电视节目。中宁县新型农村合作医疗试点进展顺利，公共卫生服务水平和应急能力进一步提高。传染病和地方病防治工作得到强化，计划免疫、妇幼保健工作不断加强。严肃执行计划生育政策和法规，川区开展知情选择和优质服务活动，山区实施“少生快富”扶贫工程，人口和计划生育工作稳步推进。环境与资源保护工作成效显著。老龄和残疾人工作又有新发展。加强民兵预备役建设，支持了中卫军分区、武警部队建设，强化了军队转业干部、退役士兵安置和拥军优属工作，“双拥”共建活动深入开展。统计、物价、工商管理和档案、气象、防震减灾、人防、外事、侨务等方面的工作取得新成绩。

社会保障逐步完善，人民生活不断提高。认真落实扶持就业与再就业的各项优惠政策，努力拓宽就业渠道。全市新增就业岗位6356个，其中一次性购买“4050”人员再就业岗位340个。千方百计加大社会保障资金投入，养老、失业和医疗保险动态覆盖率有效提高。全年累计发放低保资金2468.5万元、各类救灾救济资金1905.4万元，帮助灾民建房700户2046间。实施了沙坡头移民搬迁等扶贫开发项目，脱贫人口达到4.3万人。有组织地转移农村剩余劳动力16.6万人次，实现劳务收入6.3亿元。巩固禁牧封育成果，草畜产业稳步推进。积极筹措抗旱资金，支援山区抗旱，山区群众生产生活得到极大改善。

民主法制不断加强，行政能力明显增强。自觉接受市人大及其常委会的法律监督和工作监督、市政协及各民主党派、工商联的民主监督、社会各界的群众监督和新闻媒体的舆论监督。全年办理人大代表议案5件、建议31条，政协提案182件。依法行政和政务公开工作稳步推进。实行市发改委、经委、财政局等10个部门合署办公，有效提高了政府工作效率。建成了中卫市政府网站。加强经营性土地使用权出让招拍挂，加大审计监督和行政监察力度，查处各类违纪案件22件，结案22件，政府系统廉政建设取得新成效。深入开展普法教育，公民法律意识进一步增强。加大劳动监察力度，清欠农民工工资1253万元。重视和加强信访工作，有效化解了各类矛盾纠纷。民族宗教工作稳步推进。加强安全生产监督管理，强化公众聚集场所安全，预防道路交通事故，开展重点行业和领域的安全生产专项整治,全年无特大事故发生。完善治安防控体系建设，开展“严打”整治行动，社会治安状况良好。

各位代表！

过去一年取得的成绩来之不易，这是自治区党委、政府和市委正确领导的结果，是市人大、市政协及社会各界监督支持的结果，是全市各族人民共同努力的结果，是驻卫各单位、部队积极帮助的结果。在此，我代表市人民政府，向全市工人、农民、知识分子、干部、解放军指战员、武警官兵、公安干警，表示崇高的敬意！向人大代表、政协委员、各民主党派、工商联、人民团体、离退休老同志，向关心和支持中卫发展的社会各界人士，表示衷心的感谢！

在肯定成绩的同时，我们也应清醒地看到，全市经济社会发展中还存在着一些矛盾和问题。主要是：经济发展和城市建设中的资金、土地等生产要素供需矛盾还比较突出；企业自主创新能力还不强；农业产业化、标准化程度还不高；各项社会事业发展还不够协调；发展环境尤其是软环境还不尽人意；政府部门作风和效能建设有待增强，服务质量还需提高。对于这些困难和问题，我们将采取更加有力的措施，认真加以解决。

二、 “十一五”发展的指导思想和战略任务

“十一五”时期，是我市全面建设小康社会和加快现代化建设的关键时期。综观全局，我市经济发展机遇和挑战并存，机遇大于挑战。当前国际产业资本加速向我国转移，沿海发达地区相关产业加速向中西部地区转移，这为我们立足于区位、交通、资源等综合优势，谋求联动发展，提供了广阔前景。国家中部崛起战略的实施，对自主创新的政策支持，为我们提供了难得机遇。全市经济积蓄的能量持续释放，为我们加快发展创造了良好条件。凝心聚力，抓住机遇，只争朝夕，乘势而上，是我们义不容辞的责任，也是全市人民的共同愿望。

围绕建设生态旅游文化城市的目标，我们将着力实施工业强市、农业稳市、城建兴市、旅游活市、生态立市、依法治市六大战略。“十一五”期间，全市地区生产总值达到120亿元，年均增长12%，其中：第一产业18亿元，年均增长4%，第二产业66亿元，年均增长18%，第三产业36亿元，年均增长11%。地方财政收入达到6亿元，年均增长14%。全社会固定资产投资累计达到400亿元，年均增长20%。城镇居民人均可支配收入达到11000元，年均增长8.8%。农民人均纯收入达到3400元，年均增长6.3%。城镇登记失业率控制在4.5%以内。人口自然增长率控制在12.2‰以内。

“十一五”期间，全市经济社会发展的指导思想是：以邓小平理论和“三个代表”重要思想为指导，坚持以科学发展观统领经济社会发展全局，认真贯彻党的十六届五中全会精神，坚定不移地推进工业化、城市化、农业产业化和旅游特色化，大力调整优化经济结构,加快转变经济增长方式，积极发展循环经济，着力培育优势特色产业，搞好社会主义新农村建设，努力构建和谐社会，促进全市国民经济和社会各项事业更快更好地发展。

“十一五”期间，全市经济社会发展的总体构想是：

──努力建设经济繁荣的新中卫。经过五年的努力，使我市的综合经济实力和可持续发展能力有较大增强。经济结构趋于协调，基本建立起适应市场经济发展要求的新型产业体系，经济增长的质量和效益显著提高，建设经济强市的步伐明显加快。

全力实施工业强市战略。以实施造纸、电石、PVC、钢铁“四个百万吨工程”为重点，加速推进新型工业化，力争工业增加值年均增长20%以上。加快发展非公有制经济，力争非公有制经济产值在全市工业总产值中的比重占到90%以上。改造提升传统产业，大力发展高新技术产业，把中卫建成宁夏重要的冶金化工、建筑建材产业基地和西北重要的造纸、酿酒、机械制造、农副产品加工基地。充分利用国内外两种资源、两个市场，大力招商引资，谋划建设一批推动结构调整和产业升级的大项目，着力培植一批竞争实力强、经济效益好的优势骨干企业群体，真正走出一条科技含量高、经济效益好、资源消耗低、环境污染少、人力资源优势得到充分发挥的新型工业化路子。

全力实施农业稳市战略。按照生产发展、生活宽裕、乡风文明、村容整洁、管理民主的要求，坚持从实际出发，尊重农民意愿，扎实稳步推进新农村建设。坚持多予、少取、放活，加大对农业和农村的投入力度，扩大公共财政覆盖农村的范围，建立以工促农、以城带乡的长效机制。坚持用工业化理念发展农业，力争农业增加值年均增长5%左右。在稳定提高粮食综合生产能力的同时，加快发展农业优势特色产业，搞好产品深度开发和农业标准化生产，延伸产业链条，提升农业整体素质和效益，把中卫建成“世界枸杞之都”、国家级无公害设施蔬菜示范基地、“中国硒砂瓜第一市”和西部最大的商品鸡生产养殖基地。大力推进扶贫开发，发展劳务经济，转移农村剩余劳动力，做大做强劳务产业，多渠道增加农民收入，培养新农民，建设新农业，服务新农村。

全力实施旅游活市战略。打响沙坡头品牌，打造黄河漂流、沙漠探险、古刹建筑、生态观光、回乡风情、登山攀岩六大精品，建设沙坡头、寺口子、边关沙漠湿地、枸杞观光园、南华山等十大景区，把中卫建成国内外知名的旅游胜地、中国优秀旅游城市。到2010年，年接待旅游人数达到150万人次，年旅游总收入达到3.6亿元。大力发展现代服务业等第三产业，全社会消费品零售总额达到26亿元，年均增长11.9%。

不断加强基础设施建设。认真抓好事关全市经济社会发展重大项目的实施。积极配合太中（银）铁路、沙坡头北干渠等重点项目建设，做好沙坡头支线机场建设前期工作。加快建设、改造提升中卫城区、中宁、海原境内国省干道和高速公路，形成东进西出的国省干道和高速公路网络，提高农村公路通达深度和覆盖面，实现乡乡通油路、村村通等级公路的目标，构筑起连接城乡、覆盖全市的农村公路网络。

──努力建设环境优美的新中卫。经过五年的努力，使我市城市面貌有较大改观，城镇化水平达到41%，形成以市区为中心、各县城和中心镇为基础的特色鲜明、规模合理、布局科学、功能互补的城镇体系，把中卫建成西北最美丽的滨河城市。

到2010年，市区面积达到32平方公里，人口达到20万。拉大城市框架，突出新区建设，加快旧城改造，配套基础设施，加快以道路、供排水、电力、通信为重点的新区基础设施建设，形成贯通东西、连接南北、便捷通畅的城市路网和配套完善的供排水、供热、供气管网。加快以公共绿地、庭院绿化、环城林带为重点的生态园林建设。实施好市区美化、绿化、亮化工程，搞好市区沿河生态综合治理和休闲旅游项目的规划与开发。市区人均公共绿地达到10平方米，绿化覆盖率达到20%以上。加快发展城市经济，努力建设以电子信息、精细化工、商贸服务等为主导产业的现代中等城市。加快中宁、海原县城建设。加速发展一批工矿型、商贸型、旅游型等各具特色的小城镇。进一步完善城市管理手段，强化城市综合执法，切实提高城市管理水平。坚持排污许可证制度，实行污染物排放总量控制，开展油烟、烟尘、噪音污染等专项整治，实现节约发展、清洁发展、安全发展和可持续发展，争创国家级园林城市、卫生先进城市和国家环境保护模范城。

──努力建设人民富裕的新中卫。经过五年的努力，使我市城乡居民收入实现较快增长，生活质量和健康水平明显提高。

不断改善居民生活条件，全市城镇居民人均住房面积达到30平方米以上。固定电话基本普及，移动电话使用率显著提高。鼓励劳动者自主创业和自谋职业，促进多种形式就业，城镇每年新增就业4000人。建立健全与经济发展水平相适应的社会保障体系。完善城镇职工基本养老和基本医疗、失业、工伤、生育保险制度。增加财政的社会保障投入，多渠道筹措社会保障基金。发展企业补充保险和商业保险。认真解决进城务工人员社会保障问题。推进机关事业单位养老保险制度改革。完善优抚保障机制和社会救助体系。探索建立农村最低生活保障制度。加强扶贫开发，切实改善少数民族地区和山区群众的生产生活条件。着力解决关系群众切身利益的突出问题，城乡困难群众的基本生活得到有效保障。落实安全生产责任制，严格安全执法，有效遏制重特大事故。加强交通安全监管，减少交通事故。重视各种自然灾害预测预报，提高防灾减灾能力。强化对食品、药品、餐饮卫生等的监管，保障人民群众健康安全。

──努力建设社会和谐的新中卫。经过五年的努力，使我市社会事业有较大发展，人民的思想道德和科学文化素质显著提高，民主法制进一步加强，人与自然和谐相处的社会氛围初步形成。

坚持教育优先发展。巩固提高城区和中宁“两基”成果，完成海原“两基”攻坚，加强高中教育，大力发展职业教育和民办教育，建成中卫职业技术学院。加快科学技术创新和跨越，加速科技成果转化。实施人才强市战略，建立多层次的人才队伍。推行新型农村合作医疗制度，加快卫生体系建设，提高疾病预防控制和医疗救治服务能力，有效控制传染病，注重防治职业病、地方病。控制人口数量，提高人口素质，综合治理出生人口性别比。加快发展文化事业和文化产业。加大政府对文化、体育事业的投入，结合新区建设，高起点规划建设科技馆、文化馆、图书馆、博物馆、体育场（馆）和青少年活动中心、老干部活动中心、职工活动中心等一批文化体育设施，逐步形成覆盖全社会的比较完备的公共文化体育服务体系。繁荣新闻出版、广播电视、文化艺术，创造更多更好适应人民群众需求的优秀文化产品。深入开展精神文明创建和“双拥共建”活动。加强青少年思想道德教育，积极营造有利于未成年人健康成长的社会氛围。推进基层民主政治建设，完善居民自治、村民自治和各项公开办事制度，保障广大群众行使各项民主权利。深入开展普法教育，增强全民学法、用法、守法的自觉性。认真落实党的民族政策，依法加强对宗教事务的管理，巩固发展平等、团结、互助的社会主义民族关系。继续实施退耕还林还草、天然林保护、城镇大环境绿化、小流域治理、生态经济林和造纸原料林等项目建设，有效改善生态环境。大力发展循环经济，加大环境保护力度，加强农村面源污染治理，使节约资源、保护环境成为全社会的自觉行为，推进经济发展与人口、资源、环境相协调，建设资源节约型、环境友好型社会。认真实施《信访条例》，加强和改进信访工作。开展创建“平安中卫”活动，完善公共安全体系，建立完善社会预警体系和应急处理机制，发挥中卫军分区、武警部队和驻卫部队在应急处理、抢险救灾中的重要作用，提高公共安全和处置突发事件的能力。重视社会稳定工作，切实解决损害人民群众利益的突出问题。

各位代表，蓝图宏伟壮丽，任务光荣艰巨。实现“十一五”规划的目标，需要全市上下共同努力，需要广大干部群众艰苦奋斗。只要我们团结奋进、锐意进取，我们的目标就一定能够实现！

三、2006年主要工作

各位代表，2006年是实施“十一五”规划的开局之年，更是我市贯彻落实科学发展观，实现快速发展、科学发展的重要一年。切实做好今年的工作，开好局，起好步，对于鼓舞士气，加快发展，具有十分重要的意义。

今年，政府工作的主要预期目标是：全市地区生产总值65亿元，增长12%。其中：第一产业15.1亿元，增长4 %；第二产业30.9亿元，增长16%；第三产业19亿元，增长11%。全市地方财政收入3.5亿元，可比增长10.1 %；其中市本级财政收入2亿元，可比增长14.1%。全社会固定资产投资65亿元，增长20%。城镇居民可支配收入7800元，增长8.3%。农民人均纯收入2630元，增长5.2%。城镇登记失业率控制在4.5%以内。人口自然增长率控制在12.5‰以内。

实现今年经济社会发展的预期目标，必须以邓小平理论和“三个代表”重要思想为指导，认真贯彻党的十六大和十六届五中全会及市委一届四次全会精神，全面落实科学发展观。按照已确定的工作思路和工作格局，积极培育优势特色产业，加快转变经济增长方式，加速推进新型工业化、城市化、农业产业化和旅游特色化，推动全市经济社会全面、协调、可持续发展和社会和谐稳定。

重点抓好七个方面的工作：

（一）提高企业自主创新能力，推进产业结构优化升级

鼓励自主创新。通过多种形式，推动思想解放，使创新成为全社会共同价值取向，让一切有利于创业的思想进一步活跃起来，把各类创业的主体进一步激活起来，使一切领域的潜能进一步发挥起来，兴起全民创业的热潮。广泛动员城乡群众不拘形式，不拘规模，自主创业。以开放的视野，开明的政策，面向社会，面向各类人才，鼓励、引导和支持能人创办、领办各类企业。

培优培强优势产业。围绕造纸、酿酒、建筑建材、冶金化工、农副产品加工、机械制造等支柱产业，做大做强美利纸业、宁夏红集团、秦毅集团、赛马集团中宁公司、赢嘉实业、赢海集团、大河机床等一批优势骨干企业，提高企业技术创新能力，培育科技人才队伍，推进产业结构优化升级。通过实施造纸、电石、PVC、钢铁“四个百万吨工程”，加快上下游产品配套，形成企业集聚、产业集群，打造产业特色。全年开工建设工业项目72个。重点抓好美利纸业30万吨涂布白卡纸、10万吨杨木化机浆、2台5万千瓦热电机组，宁夏红集团2万吨枸杞饮料、1万吨枸杞酒、5000吨葡萄酒，昊丰伟业年产30万吨生铁，大河机床年产300台数控珩磨机床等项目建设。全市工业增加值增长20 %，利税增长15%。

加速园区经济发展。完善美利、宁新工业园区及常乐、镇罗、宣和等工业基地基础设施，硬化美利工业园南区、镇罗和常乐工业基地主干道路。放大美利、宁新工业园区初步形成的集聚效应，做大做强园区经济。把招商引资作为经济工作的“一号工程”，采取多种形式，吸引更多的客商来卫投资兴业，力争全年招商引资实际到位资金达到15亿元。

完善企业改制和管理。围绕建立现代企业制度和完善的公司法人治理结构，继续深化以产权制度改革为主要内容的企业改革，加快改制企业职工身份置换。进一步深化企业内部用工、人事和分配制度改革。规范企业经营行为，加强企业资金、技术、人才、营销、质量、安全管理，以人为本，关爱员工。实施名牌战略，推进企业标准化生产，品牌立市，规模占市，提高企业的市场竞争能力。

大力发展循环经济。推进节能、节水、节地、节材，加强资源综合利用，建立完善再生资源回收利用体系，积极推行清洁生产，形成低投入、低消耗、低排放和高效率的节约型增长方式。鼓励开发和应用节能降耗的新技术、新工艺，加强环境保护，逐步淘汰高能耗、高物耗的设备和产品。

（二）切实加强“三农”工作，努力建设社会主义新农村

推进现代农业建设。稳定提高粮食综合生产能力，加快发展农业优势特色产业。新增枸杞2.5万亩，累计达到18.6万亩。新增压砂地20万亩，累计达到70万亩；新增设施蔬菜1.5万亩，累计达到6.8万亩，建成三个集中连片的无公害设施蔬菜基地。抓好宣和百万只鸡养殖园区、永康百万只鸡养殖园区东区的达产达标；启动无规定疫病区建设，提升园区综合管理水平；推行“小规模、大群体”养殖模式，发展以10—20万只规模为单元的养鸡园区10个，全市鸡饲养量达到1800万只。继续推进草畜、生猪、优质米、马铃薯产业快速发展。切实做好动物疫病防治，确保不发生重大疫情。加快农业产业化经营步伐，吸引大企业、大集团参与农副产品精深加工；发展农村专业合作经济组织，组建流通额300万元以上的专业合作社、协会8家；建设和完善大中型农产品专业批发市场5个；推进农业标准化生产，落实无公害和绿色农产品生产标准，打响绿色品牌，提高农产品的市场占有份额。

大力发展农村公共事业。加大农村基础设施建设投入，加强灌区灌溉管理、农村安全饮水、小流域综合治理工作。强化乡村道路建设和管理，硬化永康养殖园区主干道路，完成中沟路、宣东路两条30公里三级油路和香山地区景庄至下河沿55公里三级砂砾路建设，年内新建、硬化乡村道路200公里以上，累计达到500公里。继续实施“塞上农民新居”工程，完成10个以上庄点砖混、砖木结构住房建设任务，配套照明、供排水、绿化等设施，带动全市村庄环境整治，逐步改善村容村貌。大力普及农村沼气，积极发展适合农村特点的清洁能源。继续实施农村广播影视公共服务体系建设项目，使城区香山和海原兴仁群众免费收看到8套电视节目。加快生态环境建设，实施好香山硒砂瓜引黄补灌、沙坡头北干渠城区段连通等项目，巩固退耕还林、退牧还草、封育禁牧成果，改善山川生态环境。

千方百计增加农民收入。充分挖掘农业内部增收潜力，扩大养殖、园艺等劳动密集型产品和绿色食品的生产，努力开拓农产品市场。实施避灾抗旱战略，发展山区特色经济。加快发展劳务经济，加强农村劳动力技能培训，引导农村剩余劳动力向非农产业和城镇有序转移，带动乡镇企业和小城镇发展，海原及城区、中宁山区每个家庭至少输出一名劳动力，力争全年劳务输出13.6万人。加大扶贫开发力度，实施整村推进工程，搞好城区1个村、中宁3个村、海原23个村的整村推进工作，年内安置移民1万人，实现脱贫2.3万人。

（三）强化基础设施建设，加速推进城镇化进程

突出规划的龙头作用。高品位规划老城区，高起点设计新城区，扩大新区控制性详规覆盖面，使规划建设的每一个建筑都经得起历史考验。严格规划管理，坚决查处建筑的违规行为，确保规划的严肃性。

加快市区建设步伐。开工建设行政中心前后广场、环城东路、环城西路、中央大道中段南路改造及新区次干道，完成滨河大道二期工程12.5公里二级油路、迎湖公路12.3公里二级油路建设，初步形成内连外接、环网互补的道路骨架。加快建设行政中心、新闻中心等单位办公楼，抓好新区供排水、供热、通信、商贸、医疗等基础设施建设，完善新区服务功能。实施中央大道等六条主干道路绿化工程，加快建设黄河湿地公园、新区生态湖、百万亩环城生态防护林带等环境工程，扩大城市绿化覆盖率。实施“六街”（长城街、文昌北街、应理北街、香山街、商业南北街）、“四巷”（槐树巷、幼儿园巷、鼓楼南街一道巷和二道巷）改造工程，美化、亮化旧城街道。抓好官桥花园、东方家园等旧城改造项目，完成建筑面积30万平方米以上。

加快县城和小城镇建设。抓好中宁、海原县城规划，体现特色，稳步推进。加快中宁县城集污及污水处理厂续建、枸杞文化博物馆、县城供水改扩建、大庆路、枸杞花园工程续建等项目和海原县城改造扩建步伐。因地制宜，重点发展一批工矿型、交通枢纽型、商贸型等中心小城镇。

提升城市经营管理水平。建立完善城市建设投融资体制，逐步形成多形式、多元化、多渠道的投融资机制。强化基础设施的资产观念、市场观念、经营观念和效益观念，将各类城市有形和无形资产全部推向市场，盘活存量。加强政府对土地市场的宏观调控，防止土地收益流失。强化城市管理，开展专项市容整治活动，集中整治占道经营、“五小车辆”乱停乱放、户外广告、城市违章建筑、卫生垃圾等，规范居民行为，优化人居环境。

（四）加快发展以旅游为重点的现代服务业，积极扩大消费需求

突出抓好旅游业。搞好沙坡头生态风情苑、寺口子、中宁枸杞园、海原环球大震遗址等重点景区景点开发建设。引进大企业、大集团参与开发旅游资源，改善基础设施，提高管理水平。整顿旅游市场，规范旅行社经营行为。办好“中国宁夏（沙坡头）大漠·黄河国际旅游节”、首届宁夏中卫硒砂瓜节等节事活动，创新宣传营销手段，多渠道、多途径宣传推介我市旅游产品和项目，提高中卫知名度。年内，全市旅游接待人数达到95万人次，旅游总收入达到2.2亿元，直接从业人员达到2000人。

搞活商贸流通。引进培植一批现代物流企业，加快中卫市场、正丰商贸中心等商贸项目建设。加速推进金融、保险、房地产、信息等其他各业，促进第三产业快速发展。严厉打击制售假冒伪劣商品的违法行为，维护良好的市场秩序。全社会消费品零售总额达到16.5亿元，增长10.7%。

（五）进一步做好财税金融工作，确保财政收入稳定增长

坚持依法治税，强化税收征管，积极推行社会综合治税，切实做到应收尽收。进一步拓展财政增收渠道，积极争取中央、自治区财政转移支付、专项资金和国家开发银行信贷支持。调整优化财政支出结构，减少一般性支出，加大对农业、社会保障和教育、卫生等公共事业的支持力度，促进城乡协调发展和经济社会可持续发展。加快实施农村综合改革试点，建立新型镇乡财政管理体制。全面深化部门预算和国库集中收付改革，强化财政专项资金管理和企事业单位国有资产监督管理。加强信用体系建设，营造良好的金融生态发展环境。切实支持金融部门工作，鼓励金融机构加大信贷投入，优化信贷结构，防范和化解金融风险，为经济和社会发展提供有力的金融支持。

（六）加强和谐社会建设，不断提高人民生活水平

切实做好就业和社会保障工作。加快建立政府扶助、社会参与的职业技能培训机制。完善对困难群众的就业援助制度，建立促进扩大就业的有效机制，不断增加就业岗位，完善中介服务，加强就业培训，鼓励自主创业。完善城镇职工基本养老和基本医疗、失业、工伤保险制度。积极探索进城务工人员社会保障问题。重视保障妇女儿童权益。做好弱势群体的法律援助工作。积极发展残疾人事业。加强社会福利事业建设，完善优抚保障机制和社会救助体系，支持社会慈善、社会捐赠、群众互助等社会扶助活动。

发展教育、科技、文化、卫生等事业。坚持教育优先发展，进一步整合教育资源，全面实施素质教育，切实提高教育教学质量，加大教育投入，建立有效的教育资助体系。对农村义务教育阶段学生全部免收学杂费。大力发展职业教育和特殊教育，建设高等职业技术学院，迁建中卫中学、中卫六小。加快科学技术创新，努力提高原始创新、集成创新和引进消化吸收再创新能力。加大人力资源开发投入，推进市场配置人才资源。继续推进文化体制改革，不断发展文化体育事业和文化产业，加强城乡、社区文化体育设施建设，规划建设文化馆、图书馆、博物馆、体育场（馆）。加强文化市场管理，营造扶持健康文化、抵制腐朽文化的社会环境。大力开展全民健身运动，提高竞技体育水平。强化农村卫生基础设施建设，实施城区和中宁县新型农村合作医疗试点，完善公共卫生和医疗服务体系，提高疾病预防控制和医疗救治服务能力。加强妇幼卫生保健，发展社区卫生服务。加大计划生育综合改革力度，深化优质服务工作，提高出生人口素质。进一步做好防震减灾、气象、统计、人防、档案、地方志等工作。

加强社会主义精神文明建设和民主法制建设。推进文明社区、文明村镇、文明行业建设，倡导科学、健康、文明、向上的生活方式。抓好未成年人的思想道德建设，构建学校、家庭、社会“三位一体”的教育体系。加强国防后备力量建设，深入开展国防教育，增强国防意识，支持军队建设，巩固“双拥”成果，努力创建全国“双拥模范城”。继续推进政务公开，不断发展基层民主。巩固和壮大最广泛的爱国统一战线。加强与工会、共青团、妇联等人民团体的联系。进一步做好民族、宗教和侨务工作。全面落实行政执法责任制，大力推进“依法治市”进程。做好普法工作，切实增强公民学法、用法、守法的自觉性。

积极促进社会和谐。落实安全生产责任制，进一步抓好安全生产工作，有效遏制重特大事故。强化对食品、药品、餐饮卫生等的监管，保障人民群众健康安全。妥善处理不同利益群体关系，认真解决人民群众最关心、最直接、最现实的利益问题。加强和谐社区、和谐村镇建设，增强社会和谐基础。正确处理新形势下的人民内部矛盾，畅通诉求渠道，完善社会利益协调和社会纠纷调处机制。建立健全社会预警体系和应急救援机制，提高处置突发性事件能力。加强社会治安综合治理，深入开展“平安中卫”创建活动，依法严厉打击邪教等各种犯罪活动，破除封建迷信，维护社会稳定，保障人民群众安居乐业。

（七）加强政府自身建设，不断提高行政能力

全面推进依法行政，努力建设法治政府。认真实施《行政许可法》和国务院《全面推进依法行政实施纲要》，全面推行行政执法责任制，严格依照法定的权限和程序行使权力、履行职责、接受监督。加强政风、行风建设，完善和深化行风评议制。改革和完善行政决策机制，不断提高决策的民主化和科学化水平。自觉接受同级人民代表大会及其常委会和政协的监督，主动接受新闻媒体和社会公众的监督。坚决执行人大的各项决议，认真办理人大代表议案、建议和政协提案。主动听取各民主党派、工商联和社会各界的意见和建议。支持审判和检察机关依法独立行使审判权和检察权。扩大政务公开范围，增强政府工作的透明度和公信力。

加强反腐倡廉工作，努力建设清廉政府。建立健全教育、制度、监督并重的惩治和预防腐败体系，努力从源头上预防和治理腐败。坚持从严治政，筑牢警示训诫防线。坚决纠正征地拆迁、企业改制中损害群众利益的不正之风。严肃查处干扰破坏投资环境的人和事，以清廉严明的形象取信于民。

切实改进工作作风，努力建设务实政府。各级政府要全面落实科学发展观和正确政绩观，把主要精力集中在干实事、求实效上。围绕群众关注的热点难点问题，深入开展调查研究，及时发现和解决问题。加强督促检查，严格考核奖惩。上下级之间要相互支持，部门之间要密切配合，形成抓落实的强大合力，确保各项目标任务的圆满完成。

加快转变政府职能，努力建设高效政府。进一步推进政企分开、政资分开、政事分开、政府和市场中介组织分开，减少和规范行政审批。加强政府经济调节、市场监管、社会管理和公共服务职能，着力创造良好发展环境，为投资商和企业提供方便优质高效的服务。实行编制实名制管理，进一步做好人事管理工作。加强公务员的学习和培训，努力建设一支人民满意的公务员队伍。

各位代表！时代赋予我们重任，人民寄予我们厚望。喜看今朝，全市人民正谱写建设经济强市的壮丽篇章；展望未来，中卫大地将呈现构建和谐社会的美好前景。让我们高举邓小平理论和“三个代表”重要思想的伟大旗帜，在自治区党委、政府和市委的领导下，解放思想，抢抓机遇，开拓创新，务实苦干，为建设经济繁荣、社会和谐、环境优美、人民富裕的新中卫而努力奋斗！
